# A statistical analysis method for probability distributions in Erdös–Rényi random networks with preferential cutting–rewiring operation

**DOI:** 10.3389/fnetp.2024.1390319

**Published:** 2024-10-17

**Authors:** Yu Qian, Jiahui Cao, Jing Han, Siyi Zhang, Wentao Chen, Zhao Lei, Xiaohua Cui, Zhigang Zheng

**Affiliations:** ^1^ College of Physics and Optoelectronic Technology, Baoji University of Arts and Sciences, Baoji, China; ^2^ School of Systems Science, Beijing Normal University, Beijing, China; ^3^ Institute of Systems Science, Huaqiao University, Xiamen, China; ^4^ College of Information Science and Engineering, Huaqiao University, Xiamen, China; ^5^ School of Mathematical Sciences, Huaqiao University, Quanzhou, China

**Keywords:** network physiology, biological science, brain networks, complex systems, network models

## Abstract

The study of specific physiological processes from the perspective of network physiology has gained recent attention. Modeling the global information integration among the separated functionalized modules in structural and functional brain networks is a central problem. In this article, the preferentially cutting–rewiring operation (PCRO) is introduced to approximatively describe the above physiological process, which consists of the cutting procedure and the rewiring procedure with specific preferential constraints. By applying the PCRO on the classical Erdös–Rényi random network (ERRN), three types of isolated nodes are generated, based on which the common leaves (CLs) are formed between the two hubs. This makes the initially homogeneous ERRN experience drastic changes and become heterogeneous. Importantly, a statistical analysis method is proposed to theoretically analyze the statistical properties of an ERRN with a PCRO. Specifically, the probability distributions of these three types of isolated nodes are derived, based on which the probability distribution of the CLs can be obtained easily. Furthermore, the validity and universality of our statistical analysis method have been confirmed in numerical experiments. Our contributions may shed light on a new perspective in the interdisciplinary field of complexity science and biological science and would be of great and general interest to network physiology.

## 1 Introduction

The collective behaviors that emerged on different kinds of complex systems have become the central topics under investigation since the seminal “small-world” and “scale-free” network models were successively proposed by Strogatz and Barabási (Watts and Strogatz, 1998; Barabási and Albert, 1999). Several typical types of spatiotemporal dynamical behaviors, such as synchronous phenomena (Wu et al., 2012; Walter et al., 2014; Zhang et al., 2015; Andrzejak et al., 2017; Rybalova et al., 2020; Ghosh et al., 2023), self-sustained oscillations (Roxin et al., 2004; Sinha et al., 2007; Qian et al., 2010a; Qian et al., 2010b; Isele and Schöll, 2015; Fretter et al., 2017), and chimera and chimeralike states (Hagerstrom et al., 2012; Omelchenko et al., 2013; Semenova et al., 2016; Kachhara and Ambika, 2021; Lei et al., 2022; Lei et al., 2023; Zhang H. et al., 2024), have been reported. For example, Rybalova et al. (2020) exposed the relay and complete synchronization in heterogeneous multiplex networks of chaotic maps. Sinha et al. (2007) discussed the emergence of self-sustained patterns in small-world excitable media. Kachhara and Ambika revealed the frequency chimera state induced by differing dynamical timescales (Kachhara and Ambika, 2021). Lei et al. (2023) and Zhang H. et al. (2024), respectively, uncovered the chimeralike oscillation modes on excitable scale-free networks and the alternate attractor chimeralike states on rings of chaotic Lorenz-type oscillators.

One of the most classical network models, the *Erdös–Rényi random network* (ERRN), was proposed by P. Erdös and A. Rényi (Erdös and Rényi, 1959; Erdös and Rényi, 1960) and is utilized to explore these issues. Many interesting phenomena were found, and great achievements were realized by this paradigmatic network model (Gong et al., 2005; Xu and Liu, 2008; Tattini et al., 2012; Ferrari et al., 2013; Schmeltzer et al., 2014; Almeira et al., 2020; Masoumi et al., 2022; Kartal and Kartal, 2023; Qian, 2014; Qian et al., 2017; Qian and Zhang, 2017). For example, Gong et al. discussed the synchronization of Erdös–Rényi networks (Gong et al., 2005). Tattini et al. investigated the coherent periodic activity on excitatory Erdös–Rényi neural networks and exposed the key role of network connectivity (Tattini et al., 2012). Almeira et al. (2020) discovered the scaling of percolation transitions on Erdös–Rényi networks under centrality-based attacks. Kartal et al. studied the complex dynamics of the COVID-19 mathematical model on the Erdös–Rényi network (Kartal and Kartal, 2023). Qian et al. first reported the emergence of the self-sustained oscillations on excitable Erdös–Rényi random networks and exposed the determinants (Qian, 2014; Qian et al., 2017) and then revealed the effects of time delay and connection probability on the corresponding oscillations and synchronization transitions (Qian and Zhang, 2017).

In addition to the collective behaviors that can self-organize to emerge on ERRNs consisting of different types of local units, the statistical properties of the ERRN are also an important issue. For example, Erdös and Rényi were the first to study the distribution of the maximum and minimum degree in a random graph (Erdös and Rényi, 1959), and the full degree distribution was derived later by Bollobás (1981). Chung and Lu discussed the diameter of sparse random graphs (Chung and Lu, 2001). Martin and S̆ulc (2010) investigated the return probabilities and hitting times of random walks on sparse Erdös–Rényi graphs. Bizhani et al. (2011) explored the random sequential renormalization and agglomerative percolation on Erdös–Rényi networks. Hartmann and Mézard (2018) first studied the distribution of diameters for Erdös–Rényi random graphs and then discussed the distribution of shortest path lengths of subcritical Erdös–Rényi networks (Katzav et al., 2018). However, whether the statistical properties of an ERRN with specific operation can be theoretically derived is still unknown.

Nowadays, investigating the physiological processes from the perspective of network physiology is an important topic in the interdisciplinary field of complexity science and biological science. Several excellent contributions were achieved in this field (Ivanov, 2021; Shi et al., 2022; Sinha et al., 2022; Goodfellow et al., 2022; Schöll et al., 2022; Venkadesh et al., 2024; Rosenblum, 2024; Zhang Z. et al., 2024; Qian et al., 2024a). For example, Ivanov (2021) summarized the new field of network physiology, that is, building the human physiolome. Sinha et al. (2022) discussed the perspectives on understanding aberrant brain networks in epilepsy. Schöll et al. (2022) reviewed the adaptive networks in functional modeling of physiological systems.

As we know, there exists a cost-efficiency trade-off between the physical cost of the network and the information integration among the whole system in organizing structural and functional brain networks. To save wiring costs, brain networks tend to build module structures to implement localized functions. To achieve global information integration among the separated functionalized modules, long-range synapses can be created via synaptic plasticity on these local structures. More importantly, the newly reshaped long-range synapses will preferentially connect to specific hub regions to fulfill special physiological functions among the whole brain systems. So we would ask whether the appropriate functional model can be proposed to describe this physiological process from the perspective of network physiology? We think this is an important issue of great and general interest to network physiology.

In this article, the *preferentially cutting–rewiring operation* (PCRO) is proposed to approximatively describe the above physiological process. We have found that, by applying the PCRO on the ERRN with certain preferential constraints, the topological feature of the given network will change dramatically. Then, the theoretical statistical properties of the operated ERRN are studied. The remainder of the article is organized as follows. Section 2 introduces the cutting and rewiring procedures of the PCRO. In Section 3, we apply the PCRO to the classical ERRN. The statistical analysis method proposed in Section 4 supports studying the theoretical statistical properties of an ERRN with a PCRO. The validity and the universality of our statistical analysis method are, respectively, confirmed in Sections 5, 6. Finally, we give the conclusion in the last section.

## 2 The cutting and rewiring procedures of a PCRO

**FIGURE 1:**
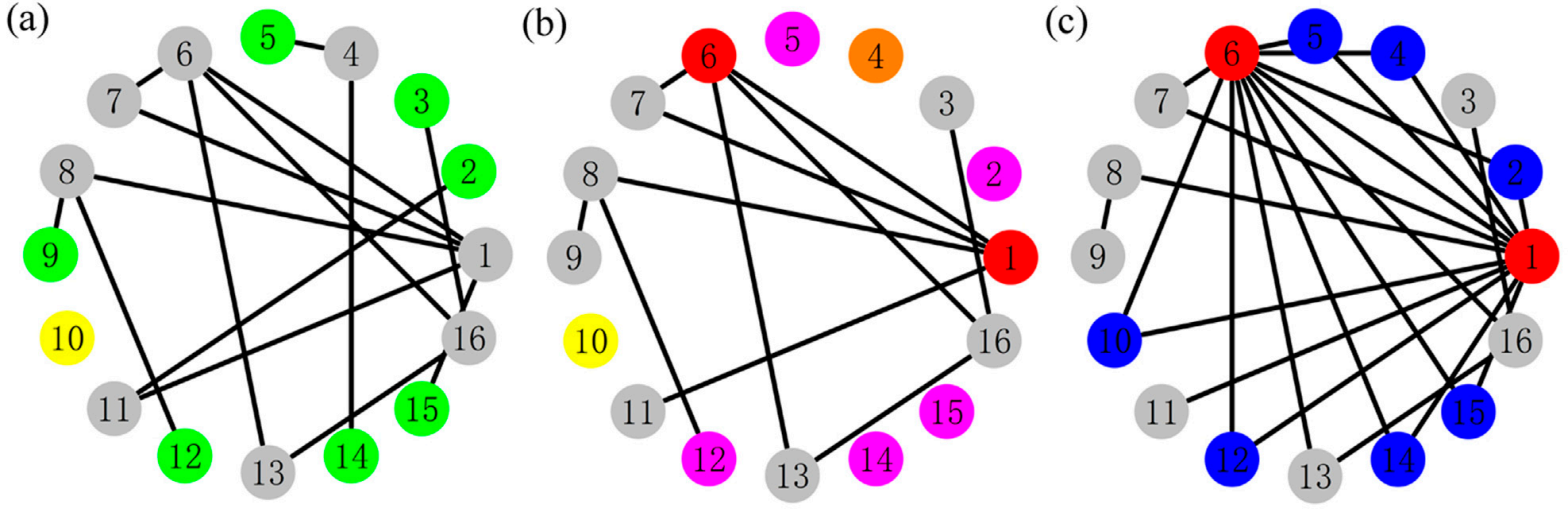
(Color online) The schematic diagram of the *preferentially cutting–rewiring operation* (PCRO) proposed in this article. Three indicators, $k_{1}$ , $k_{2}$ , and $k_{3}$ , are, respectively, utilized to represent the preferential constraints in the cutting procedure (denoted by $k_{1}$ and $k_{2}$ ) and rewiring procedure (only by $k_{3}$ ) of the PCRO. Here, a network consisting of 16 nodes is used as an example to introduce the PCRO procedures. The specific preferential constraints are selected as $k_{1}=1$ , $k_{2}={\left\langle k\right\rangle }_{AV}$ , and $k_{3}=k_{Max}^{\prime }$ . Here, ${\left\langle k\right\rangle }_{AV}$ presents the actual value of the average degree in the network before the cutting procedure, and $k_{Max}^{\prime }$ is the maximum degree in the corresponding network after the cutting procedure. **(A)** The initial structure without the PCRO. The green cells denote the nodes satisfying the preferential constraint in the cutting procedure (i.e., the nodes possessing degrees within $k_{1}\leq k_{i}\leq k_{2}$ ), which are considered candidates for performing the following cutting operation. **(B)** The network structure gained after the cutting procedure. The connections of the initially green candidates satisfying the PCRO probability condition are all discarded. The red cells represent the two nodes satisfying the preferential constraint in the rewiring procedure (i.e., the nodes possessing degree $k_{i}=k_{3}$ after the cutting procedure), which are selected as the target nodes to be rewired in the following rewiring procedure. **(C)** The network structure obtained after the rewiring procedure. The three types of isolated nodes after the cutting procedure [i.e., the yellow, pink, and orange nodes in panel **(B)**] are rewired to the two red target nodes.

In this part, we first introduce the PCRO, which is adopted to regulate the structure of a given network. The PCRO proposed in the present article contains two different stages: the cutting procedure and the rewiring procedure with specific preferential constraints, which are denoted by $k_{1}$ , $k_{2}$ (the preferential constraint indicators used in the cutting procedure) and $k_{3}$ (the constraint indicator in the rewiring procedure), respectively.The schematic diagram of the PCRO is presented in Figure 1, and the specific preferential constraints are selected as $k_{1}=1$ , $k_{2}={\left\langle k\right\rangle }_{AV}$ , and $k_{3}=k_{Max}^{\prime }$ . Here, ${\left\langle k\right\rangle }_{AV}$ presents the actual value of the average degree in the network before the cutting procedure, and $k_{Max}^{\prime }$ is the maximum degree in the corresponding network after the cutting procedure. Figure 1A displays the initial structure without a PCRO, which consists of 16 nodes. The seven green cells i=2,3,5,9,12,14, and 15 denote the nodes possessing degrees within $k_{1}\leq k_{i}\leq k_{2}$ , which satisfy the preferential constraint in the cutting procedure. Consequently, these seven green cells are considered candidates for performing the following cutting operation. To do this, we introduce the PCRO probability condition $P_{PCRO}$ to determine whether each selected candidate will be operated or not in the cutting operation. Specifically, for each green candidate, we execute the cutting operation with probability p . If the PCRO probability condition is satisfied (i.e., $p\leq P_{PCRO}$ ), all the connections of this candidate will be discarded; otherwise (i.e., $p>P_{PCRO}$ ), they will be reserved completely. The yellow cell i=10 denotes the isolated node that initially existed in the network, which is named the *naturally isolated node* (NIN). The other gray cells indicate the remainder of ordinary nodes without any operation in the cutting procedure.

By applying the cutting procedure with a specific PCRO probability $P_{PCRO}$ , the structure of the initial network will change dramatically, and the corresponding result is illustrated in Figure 1B. In the current case, the initially green candidates i=2, 5, 12, 14 , and 15 satisfy the PCRO probability condition, and their corresponding connections are all discarded. These operated candidates are called *actively deleted isolated nodes* (ADINs) and are colored pink. Meanwhile, the green candidates i=3 and 9 do not meet the PCRO probability condition; all their links are reserved, and they turn into gray ordinary nodes. Furthermore, after the cutting procedure, the originally ordinary gray cell i=4 becomes an isolated node due to its links connecting to the initially green candidates i=5 and 14, which are deleted in the cutting procedure. This type of newly generated isolated node is defined as a *passively deleted isolated node* (PDIN) and is colored orange. Moreover, the red cells i=1 and 6 present the two nodes satisfying the preferential constraint in the rewiring procedure, that is, the nodes possessing degree $k_{i}=k_{3}$ after the cutting procedure, which are selected as the target nodes to be rewired in the following rewiring procedure.

As the cutting procedure is completed, the second stage of the PCRO starts, that is, the rewiring procedure. In this stage, the above three types of isolated nodes, that is, the yellow NIN, the pink ADIN, and the orange PDIN, will be rewired to the two red target nodes. In the following discussion, these rewired nodes are called the *common leaves* (CLs) and are colored blue. Figure 1C shows the network structure after the rewiring operation, in which many blue CLs are formed between the two red target nodes. This makes the two target nodes possess relatively large degrees, which are consequently called hubs. Based on the illustrations shown in Figure 1, we can conclude that the PCRO is an effective method of regulating the structure of the given network, by which many CLs can be formed between the two hubs. The initially homogeneous network will become heterogeneous by applying the PCRO.

## 3 The ERRNs with a PCRO

The classical ERRN is utilized to test the effects of the PCRO. A classical ERRN can be constructed only based on a simple rule, that is, the connections between every pair of nodes in the ERRN are linked with a specific connection probability. In the present article, the initially homogeneous ERRN without a PCRO is composed of N=100 nodes and is constructed with connection probability $P_{ER}=0.05$ . Consequently, the total number of connections in the ERRN is expected to be $P_{ER}N\left(N-1\right)/2$ . Here, we should mention that by manipulating the connection probability $P_{ER}$ , one can produce a number of ERRNs with different properties. There are many network realizations for a given connection probability $P_{ER}$ . Furthermore, these two network structure parameters, that is, N=100 and $P_{ER}=0.05$ , will be used in the following if there are no special instructions.

**FIGURE 2:**
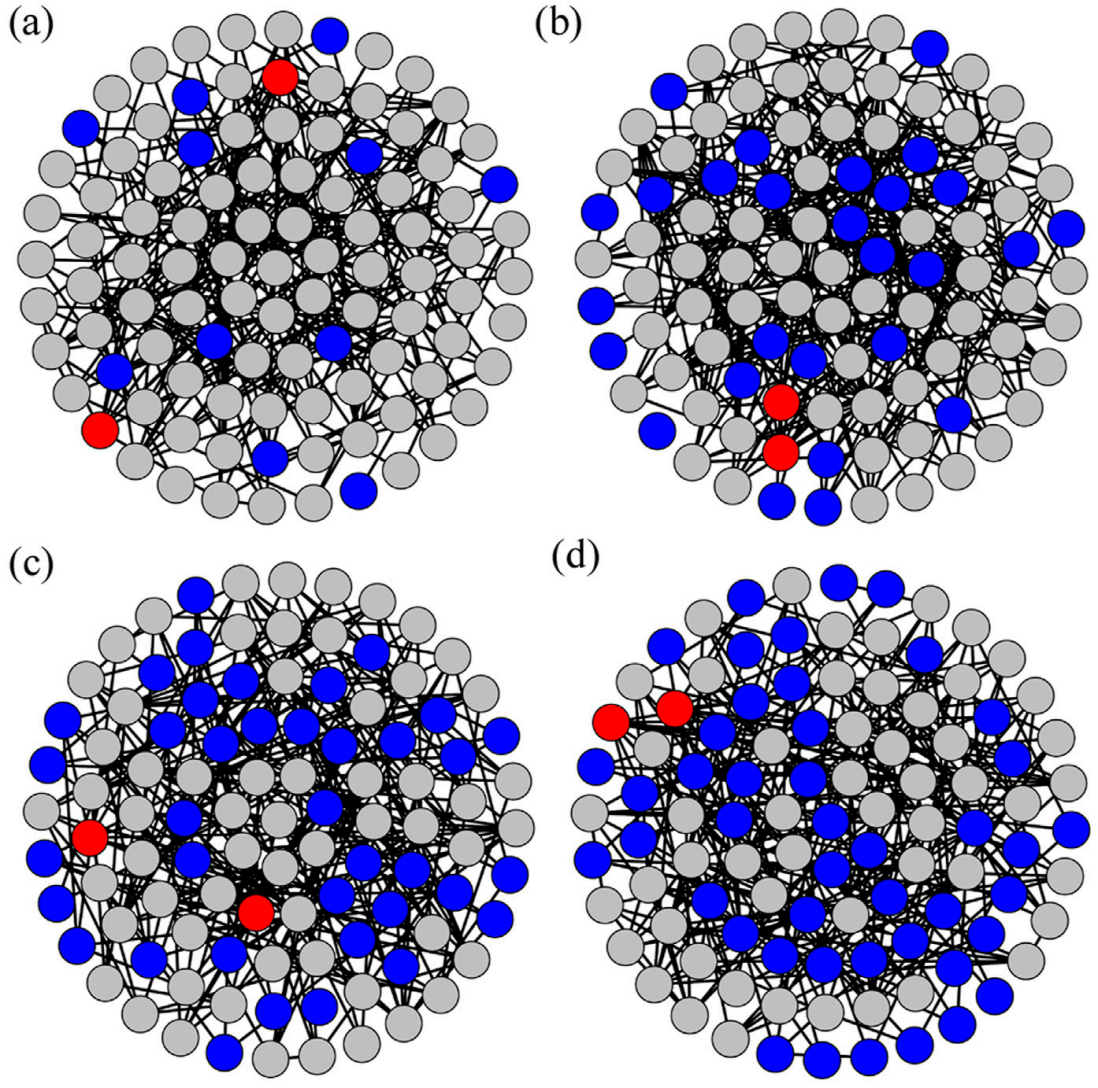
(Color online) The *Erdös–Rényi random networks* (ERRNs) obtained by the PCRO with the same preferential constraints utilized in Figure 1 (i.e., $k_{1}=1$ , $k_{2}={\left\langle k\right\rangle }_{AV}$ , and $k_{3}=k_{Max}^{\prime }$ ) for different PCRO probabilities, that is, $P_{PCRO}=0.25$ [**(A)**], $P_{PCRO}=0.50$ [**(B)**], $P_{PCRO}=0.75$ [**(C)**], and $P_{PCRO}=1.00$ [**(D)**]. The initial ERRNs without PCROs contain N=100 nodes and are constructed with connection probability $P_{ER}=0.05$ . The above preferential constraints in the cutting and rewiring procedures of PCROs and the network structure parameters utilized to construct the initial ERRNs are utilized in Figures 2–5. The red, blue, and gray circles in each panel, respectively, denote the hubs, the *common leaves* (CLs), and the ordinary cells in the operated ERRNs.

Figures 2A–D display the heterogeneous ERRNs constructed for four different parameters of the PCRO, that is, four different PCRO probabilities $P_{PCRO}=0.25$ [Figure 2A], $P_{PCRO}=0.50$ [Figure 2B], $P_{PCRO}=0.75$ [Figure 2C], and $P_{PCRO}=1.00$ [Figure 2D]. These figures clearly show three types of nodes: red hubs, blue CLs, and gray ordinary cells. Importantly, they can visualize the increasing heterogeneity of the network with increasing PCRO probability. The four network structures shown in Figures 2A–D are obtained from the initial homogeneous ERRN by a PCRO with the same preferential constraints utilized in Figure 1 (i.e., $k_{1}=1$ , $k_{2}={\left\langle k\right\rangle }_{AV}$ , and $k_{3}=k_{Max}^{\prime }$ ). This further confirms that the PCRO method proposed here has the effect of regulating the structure of the given network, such that the initially homogeneous network structure becomes heterogeneous in a controlled way.

## 4 The statistical analysis method

Now, we would ask whether the statistical properties of the ERRN with a PCRO can be theoretically derived, especially the probability distribution of CL formed in the cutting and rewiring procedures. A statistical analysis method is proposed to explore this issue. As shown in Figure 1B, three types of isolated nodes exist in an ERRN with a PCRO: the yellow NIN, the pink ADIN, and the orange PDIN. The rewiring procedure of these isolated nodes with a certain preferential constraint changes them into the CLs between the two hubs. The probability distributions of NIN, ADIN, and PDIN (denoted by $P_{NIN}$ , $P_{ADIN}$ , and $P_{PDIN}$ , respectively) are the vital tasks we aim to analyze first, based on which the theoretical analysis of the probability distribution of CL (indicated by $P_{CL}$ ) can be realized. Furthermore, as these three probability distributions of the isolated nodes are related to the *node satisfying the preferential constraint in the cutting procedure* (NSPCCP) [colored in green in Figure 1A], we first give the deduction of the analytical expression of the probability distribution of the NSPCCP (denoted by $P_{NSPCCP}$ ).

### 4.1 The probability distribution of NSPCCPs

For an arbitrary ER random network with a specific system size and connection probability $\left(N,P_{ER}\right)$ , we assume that the probability for each actual value of an edge L in the network to be $p_{L}$ . So the edge distribution in the given ER random network $p_{L}$ satisfies the following binomial distribution:

$$p_{L}=\left(\begin{array}{l} \frac{N\left(N-1\right)}{2}\\ L \end{array}\right)\cdot {\left(P_{ER}\right)}^{L}\cdot {\left(1-P_{ER}\right)}^{\frac{N\left(N-1\right)}{2}-L}.$$

By calculating the statistical average for all possible L , the expectation value of average edge in the network ${\left\langle L\right\rangle }_{EV}$ can be obtained as

$${\left\langle L\right\rangle }_{EV}=\overset{\frac{N\left(N-1\right)}{2}}{\underset{L=0}{\sum }}L\cdot p_{L}=P_{ER}\cdot \frac{N\left(N-1\right)}{2}.$$

The actual value of the average degree ${\left\langle k\right\rangle }_{AV}$ for each edge L in the network obeys

$${\left\langle k\right\rangle }_{AV}=\frac{2L}{N}.$$

Similarly, for the same ER random network with $\left(N,P_{ER}\right)$ , the probability distribution for the degree k of an arbitrary node in the given ER random network $p_{k}$ can also be described by the following binomial distribution:

$$p_{k}=\left(\begin{array}{l} N-1\\ k \end{array}\right)\cdot {\left(P_{ER}\right)}^{k}\cdot {\left(1-P_{ER}\right)}^{N-1-k}.$$ (1)

By calculating the statistical average for all possible k , we can gain the expectation value of average degree ${\left\langle k\right\rangle }_{EV}$ in the network as

$${\left\langle k\right\rangle }_{EV}=\overset{N-1}{\underset{k=0}{\sum }}k\cdot p_{k}=P_{ER}\cdot \left(N-1\right).$$

This indicates that for a given ER random network with N nodes, ${\left\langle k\right\rangle }_{EV}$ is determined by the connection probability $P_{ER}$ .

We further introduce an approximation that, for a given ER random network with N nodes, there exists an equivalent connection probability $P_{ECP}$ that can lead to the expectation value of average degree ${\left\langle k\right\rangle }_{EV}$ approximatively equals to the actual value of average degree ${\left\langle k\right\rangle }_{AV}$ ; that is,

$$\langle {k\rangle }_{AV}\approx \langle {k\rangle }_{EV}.$$

With this approximation, one can conveniently obtain the relationship between the actual value of an edge L in the network and $P_{ECP}$ as

$$L\approx P_{ECP}\cdot \frac{N\left(N-1\right)}{2}.$$

In this case, $P_{ECP}$ can be considered the equivalent connection probability of an ER random network to acquire the actual value of edge L we wanted. Moreover, for each actual value of edge L , there exists a corresponding equivalent connection probability $P_{ECP}$ .

Based on the above approximation, we can apply the equivalent connection probability $P_{ECP}$ to approximatively obtain the conditional degree distribution of an ER random network with N nodes and L edges. In this case, the conditional probability for an arbitrary node in the network being the NSPCCP obeys

$$p\left(k_{1}\leq k\leq k_{2}|L\right)=\overset{k_{2}}{\underset{k=k_{1}}{\sum }}\left(\begin{array}{l} N-1\\ k \end{array}\right)\cdot {\left(P_{ECP}\right)}^{k}\cdot {\left(1-P_{ECP}\right)}^{N-1-k}.$$

Now, we assume that there exist $N_{NSPCCP}$ NSPCCPs in the given ER random network. Therefore, the conditional probability distribution of $N_{NSPCCP}$ for a given L should satisfy the following binomial distribution:

$$P\left(N_{NSPCCP}|L\right)=\left(\begin{array}{l} N\\ NSPCCP \end{array}\right)\cdot p{\left(k_{1}\leq k\leq k_{2}|L\right)}^{N_{NSPCCP}}\cdot {\left[1-p\left(k_{1}\leq k\leq k_{2}|L\right)\right]}^{N-N_{NSPCCP}}.$$

By calculating the statistical average for all possible L , the distribution of NSPCCPs in an ER random network can be derived as

$$P_{NSPCCP}=\overset{\frac{N\left(N-1\right)}{2}}{\underset{L=0}{\sum }}P\left(N_{NSPCCP}|L\right)\cdot p_{L}.$$

### 4.2 The probability distribution of ADINs

The probability distribution of ADINs can be conveniently obtained based on the $P_{NSPCCP}$ . The ADINs are those nodes satisfying the preferential constraint in the cutting procedure, and they belong to the category of NSPCCP. They are selected by the given PCRO probability $P_{PCRO}$ , and all links of these ADINs are deleted in the cutting procedure. Here, we assume that an ER random network with a given (N,L) contains $N_{NSPCCP}$ NSPCCPs, among which there exist $N_{ADIN}$ ADINs. For a given $N_{NSPCCP}$ , the conditional probability of $N_{ADIN}$ should satisfy the binomial distribution

$$P\left(N_{ADIN}|N_{NSPCCP}\right)=\left(\begin{array}{l} N_{NSPCCP}\\ N_{ADIN} \end{array}\right)\cdot {\left(P_{PCRO}\right)}^{N_{ADIN}}\cdot {\left(1-P_{PCRO}\right)}^{N_{NSPCCP}-N_{ADIN}}.$$

By calculating the statistical average for all possible $N_{NSPCCP}$ , the probability distribution of ADINs in an ER random network can be derived as

$$P_{ADIN}=\overset{N}{\underset{N_{NSPCCP}=0}{\sum }}P\left(N_{ADIN}|N_{NSPCCP}\right)\cdot P_{NSPCCP}.$$

### 4.3 The probability distribution of PDINs

We can further solve the probability distribution of PDINs with the aid of $P_{NSPCCP}$ . Here, we first explore the conditional probability distribution of PDINs in an ER random network with a given $\left(L,N_{NSPCCP}\right)$ . Consider an arbitrary node i with degree $k_{i}$ in the network. We focus on an edge connected to node j with degree $k_{j}$ . The conditional degree distribution of node j (denoted by $p_{k_{j}}$ ) obeys the following binomial distribution

$$p_{k_{j}}=\left(\begin{array}{l} N-2\\ k_{j}-1 \end{array}\right)\cdot {\left(P_{ECP}\right)}^{k_{j}-1}\cdot {\left(1-P_{ECP}\right)}^{N-k_{j}-1}.$$ (2)

Here, $P_{ECP}$ is the equivalent connection probability of obtaining an ER random network with a given L . Because of the homogeneity of the ER network, the conditional degree distributions of the other remainder $k_{i}-1$ neighbors of the i -th node (except neighbor j ) should be the same and are described by Equation 2. If all of the i -th node’s neighbors are selected by a given PCRO probability $P_{PCRO}$ and all links of these selected neighbors are deleted in the cutting procedure, the i -th node would become a PDIN. To realize this situation, the following three conditions should be satisfied simultaneously.

Condition I:

The i -th node does not belong to the ADIN and NIN categories. The conditional probability in realizing condition I $P_{{\text{CP}}_{1}}$ includes two cases. (i) When the degree of the i -th node $k_{1}\leq k_{i}\leq k_{2}$ , the i -th node is not selected in the PCRO with probability $P_{{\text{CP}}_{1}}=1-P_{PCRO}$ ; (ii) When the degree of i -th node $1\leq k_{i}<k_{1}$ or $k_{i}>k_{2}$ , $P_{{\text{CP}}_{1}}=1$ .

By combining (i) and (ii), $P_{{\text{CP}}_{1}}$ can be written by the following piecewise function:

$$P_{{\text{CP}}_{1}}=\left\{\begin{array}{cc} 1\; & 1\leq k_{i}<k_{1},\\ 1-P_{PCRO}\; & k_{1}\leq k_{i}\leq k_{2},\\ 1\; & k_{i}>k_{2}. \end{array}\right.$$

Condition II:

The degrees of the neighbor nodes {j} of the i -th node satisfy the preferential constraint in the cutting procedure, that is, $k_{1}\leq k_{j}\leq k_{2}$ . According to Equation 2, the conditional probability II $P_{{\text{CP}}_{2}}$ should satisfy

$$\begin{array}{cc} P_{{\text{CP}}_{2}} & ={\left[p\left(k_{1}\leq k_{j}\leq k_{2}\right)\right]}^{k_{i}}\\ & ={\left[\overset{k_{2}}{\underset{k_{j}=k_{1}}{\sum }}\left(\begin{array}{l} N-2\\ k_{j}-1 \end{array}\right)\cdot {\left(P_{ECP}\right)}^{k_{j}-1}\cdot {\left(1-P_{ECP}\right)}^{N-k_{j}-1}\right]}^{k_{i}}. \end{array}$$

Condition III:

All the neighbor nodes of the i -th node are selected by the given $P_{PCRO}$ , and all the links of these neighbors are deleted in the cutting procedure. The conditional probability III $P_{{\text{CP}}_{3}}$ can be calculated as

$$P_{{\text{CP}}_{3}}={\left(P_{PCRO}\right)}^{k_{i}}.$$

By multiplying the above three conditional probabilities, we can obtain the conditional probability for the i -th node being the PDIN in the case of specific $\left(L,N_{NSPCCP},k_{i}\right)$

$$p\left[i\in \text{PDIN}|\left(L,N_{NSPCCP},k_{i}\right)\right]=P_{{\text{CP}}_{1}}\cdot P_{{\text{CP}}_{2}}\cdot P_{{\text{CP}}_{3}}.$$

As the i -th node is an arbitrary element in the given ERRN, its degree distribution $p_{k_{i}}$ should also satisfy the conditional binomial distribution of Equation 2. By calculating the statistical average for all possible $k_{i}$ of the i -th node, the above conditional probability for a given $\left(L,N_{NSPCCP}\right)$ can be obtained as

$$\begin{array}{cc} & p\left[i\in \text{PDIN}|\left(L,N_{NSPCCP}\right)\right]=\overset{N-1}{\underset{k_{i}=1}{\sum }}p\left[i\in \text{PDIN}|\left(L,N_{NSPCCP},k_{i}\right)\right]\cdot p_{k_{i}}\\ & =\overset{N-1}{\underset{k_{i}=1}{\sum }}\left(P_{{\text{CP}}_{1}}\cdot P_{{\text{CP}}_{2}}\cdot P_{{\text{CP}}_{3}}\right)\cdot p_{k_{i}}=\overset{N-1}{\underset{k_{i}=1}{\sum }}\left\{P_{{\text{CP}}_{1}}\cdot p{\left[\left(k_{1}\leq k_{j}\leq k_{2}\right)\right]}^{k_{i}}\cdot {\left(P_{PCRO}\right)}^{k_{i}}\right\}\cdot p_{k_{i}}\\ & =\left[\overset{k_{1}-1}{\underset{k_{i}=1}{\sum }}1+\overset{k_{2}}{\underset{k_{1}}{\sum }}\left(1-P_{PCRO}\right)+\overset{N-1}{\underset{k_{i}=k_{2}+1}{\sum }}1\right]\cdot {\left[\overset{k_{2}}{\underset{k_{j}=k_{1}}{\sum }}\left(\begin{array}{l} N-2\\ k_{j}-1 \end{array}\right)\cdot {\left(P_{ECP}\right)}^{k_{j}-1}\cdot {\left(1-P_{ECP}\right)}^{N-k_{j}-1}\right]}^{k_{i}}\cdot {\left(P_{PCRO}\right)}^{k_{i}}\cdot p_{k_{i}}. \end{array}$$

By further calculating the statistical average for all possible L and $N_{NSPCCP}$ , the probability for an arbitrary node in the ERRN being the PDIN can be derived as

$$\begin{array}{cc} p_{PDIN} & =\overset{\frac{N\left(N-1\right)}{2}}{\underset{L=1}{\sum }}p_{L}\cdot \overset{N}{\underset{N_{NSPCCP}=1}{\sum }}p\left[i\in \text{PDIN}|\left(L,N_{NSPCCP}\right)\right]\cdot P\left(N_{NSPCCP}|L\right)\\ & =\overset{\frac{N\left(N-1\right)}{2}}{\underset{L=1}{\sum }}p_{L}\cdot \overset{N}{\underset{N_{NSPCCP}=1}{\sum }}\left[\overset{k_{1}-1}{\underset{k_{i}=1}{\sum }}1+\overset{k_{2}}{\underset{k_{1}}{\sum }}\left(1-P_{PCRO}\right)+\overset{N-1}{\underset{k_{i}=k_{2}+1}{\sum }}1\right]\\ & \cdot {\left[\overset{k_{2}}{\underset{k_{j}=k_{1}}{\sum }}\left(\begin{array}{l} N-2\\ k_{j}-1 \end{array}\right)\cdot {\left(P_{ECP}\right)}^{k_{j}-1}\cdot {\left(1-P_{ECP}\right)}^{N-k_{j}-1}\right]}^{k_{i}}\cdot {\left(P_{PCRO}\right)}^{k_{i}}\cdot p_{k_{i}}\cdot P\left(N_{NSPCCP}|L\right). \end{array}$$

Based on the above $p_{PDIN}$ , the probability distribution of PDINs in an ER random network can be easily obtained according to the binomial distribution. Here, we assume that $N_{PDIN}$ PDINs exist in the network. The probability distribution of PDINs then follows

$$P_{PDIN}=\left(\begin{array}{l} N\\ N_{PDIN} \end{array}\right)\cdot {\left(p_{PDIN}\right)}^{N_{PDIN}}\cdot {\left(1-p_{PDIN}\right)}^{N-N_{PDIN}}.$$

### 4.4 The probability distribution of NINs

Here, we discuss the last type of isolated node, that is, the NINs colored in yellow in Figure 1B. For an arbitrary ER random network with a given $\left(N,P_{ER}\right)$ , an NIN can be considered the node with degree k=0 . According to the degree distribution in the given ER random network of Equation 1, the probability that an arbitrary node in the network is a NIN can be obtained as

$$p_{NIN}=p_{k=0}=\left(\begin{array}{l} N-1\\ 0 \end{array}\right)\cdot {\left(P_{ER}\right)}^{0}\cdot {\left(1-P_{ER}\right)}^{N-1-0}={\left(1-P_{ER}\right)}^{N-1}.$$

Here, we assume that $N_{NIN}$ NINs exist in the ER random network. So the probability distribution of NINs obeys the following binomial distribution:

$$P_{NIN}=\left(\begin{array}{l} N\\ N_{NIN} \end{array}\right)\cdot {\left(p_{NIN}\right)}^{N_{NIN}}\cdot {\left(1-p_{NIN}\right)}^{N-N_{NIN}}.$$

### 4.5 The probability distribution of CLs

Thus far, the probability distributions of NINs, ADINs, and PDINs have been derived analytically. Furthermore, these three types of isolated nodes are rewired in the rewiring procedure with a certain preferential constraint and become the CLs between the two hubs. As $P_{NIN}$ , $P_{ADIN}$ , and $P_{PDIN}$ are uncorrelated with each other, the probability distribution of CLs $P_{CL}$ can be gained based on the superposition principle as

$$P_{CL}=\underset{N_{CL}=N_{NIN}+N_{ADIN}+N_{PDIN}}{\sum }P_{NIN}\cdot P_{ADIN}\cdot P_{PDIN}.$$

## 5 The validity and applicability of the statistical analysis method

In this section, we try to apply the statistical analysis method on an ERRN with a PCRO to verify the correctness of the above conclusions. The preferential constraints $k_{1}=1$ , $k_{2}={\left\langle k\right\rangle }_{AV}$ , and $k_{3}=k_{Max}^{\prime }$ , and the network structure parameters N=100 and $P_{ER}=0.05$ , which are used as the example in Figure 2, are still utilized in this part.

**FIGURE 3:**
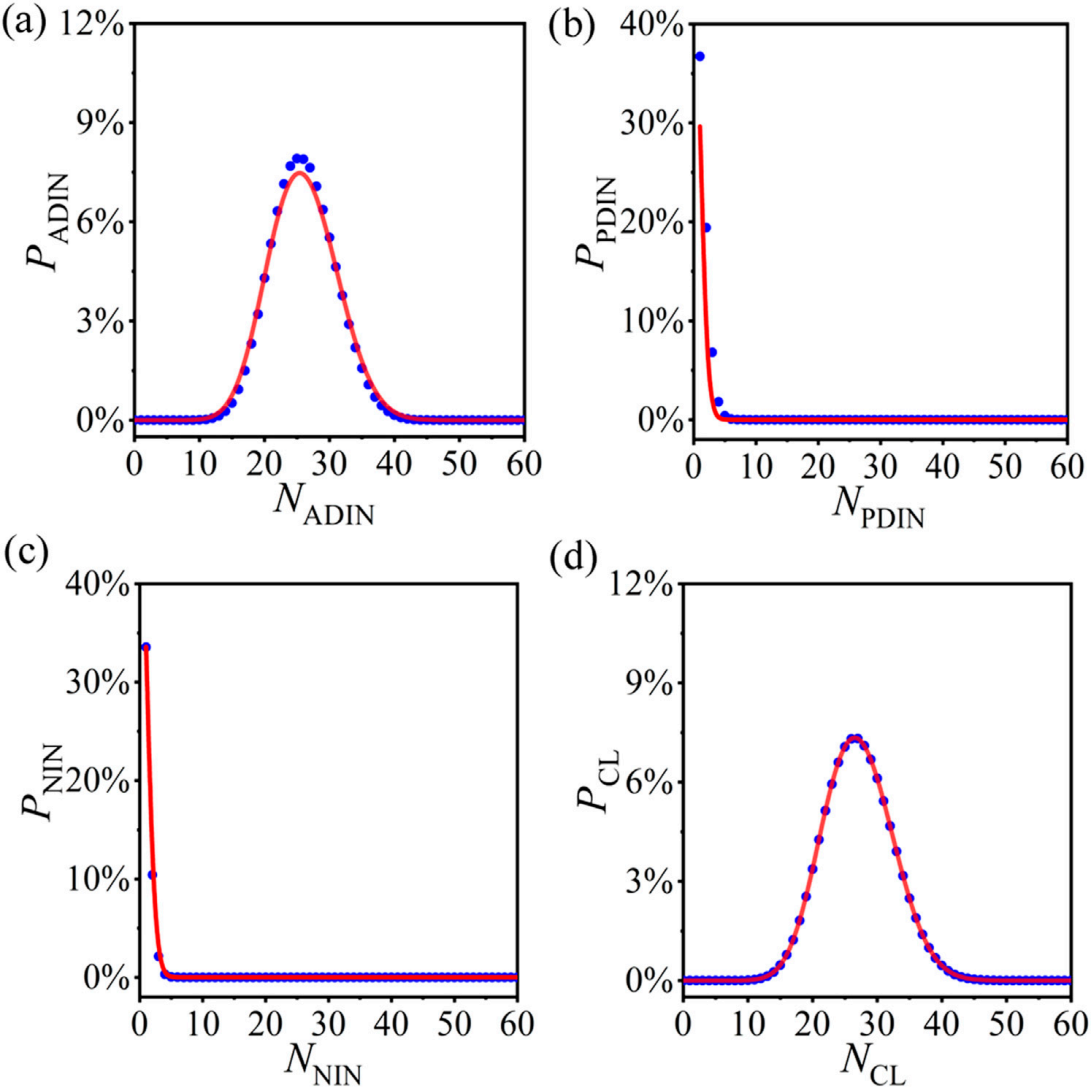
(Color online) The numerical results (blue circles) and theoretical predictions (red curves) of the probability distributions of *actively deleted isolated nodes* (ADINs) [**(A)**], *passively deleted isolated nodes* (PDINs) [**(B)**], *naturally isolated nodes* (NINs) [**(C)**], and CLs [**(D)**] in the operated ERRNs with PCRO probability $P_{PCRO}=0.50$ . In numerical simulations, $S=10^{6}$ samples are performed for each set of parameters, and this standard will also be implemented in the following figures.

We first test the probability distributions of ADINs, PDINs, NINs, and CLs in the operated ERRNs with a specific PCRO probability. The corresponding results for $P_{PCRO}=0.50$ are respectively displayed in Figures 3A–D, where the numerical simulations (blue circles) and theoretical predictions (red curves) coincide very well. In simulations, $S=10^{6}$ samples are performed for each set of parameters, and this standard will also be implemented in the following tests. The consistency of the results exposed in Figure 3 confirms the correctness of the statistical analysis method proposed in the present article.

**FIGURE 4:**
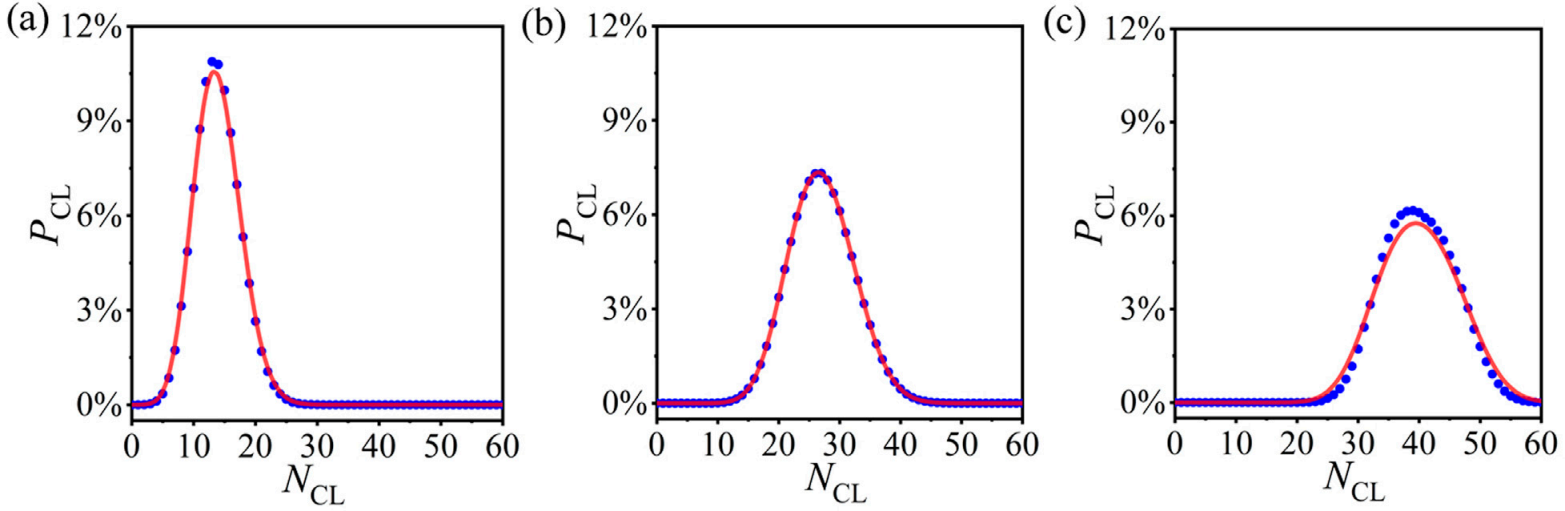
(Color online) The numerical results (blue circles) and theoretical predictions (red curves) of the probability distributions of CL in the operated ERRNs for different PCRO probabilities $P_{PCRO}=0.25$ [**(A)**], $P_{PCRO}=0.50$ [**(B)**], and $P_{PCRO}=0.75$ [**(C)**].

Now, we further verify the statistical analysis method for other PCRO probabilities. Here, we only utilize the probability distribution of CLs as the example, which is derived based on the probability distributions of ADINs, PDINs, and NINs. Figures 4A–C respectively, reveal the numerical results (blue circles) and theoretical predictions (red curves) of the probability distribution of CLs in the operated ERRNs for different PCRO probabilities $P_{PCRO}=0.25$ [Figure 4A], $P_{PCRO}=0.50$ [Figure 4B] and $P_{PCRO}=0.75$ [Figure 4C]. It is displayed explicitly in Figure 4 that, even for different PCRO probabilities, the theoretical predictions can still match well with the experimental data. This strongly confirms the validity and applicability of our statistical analysis method.

**FIGURE 5:**
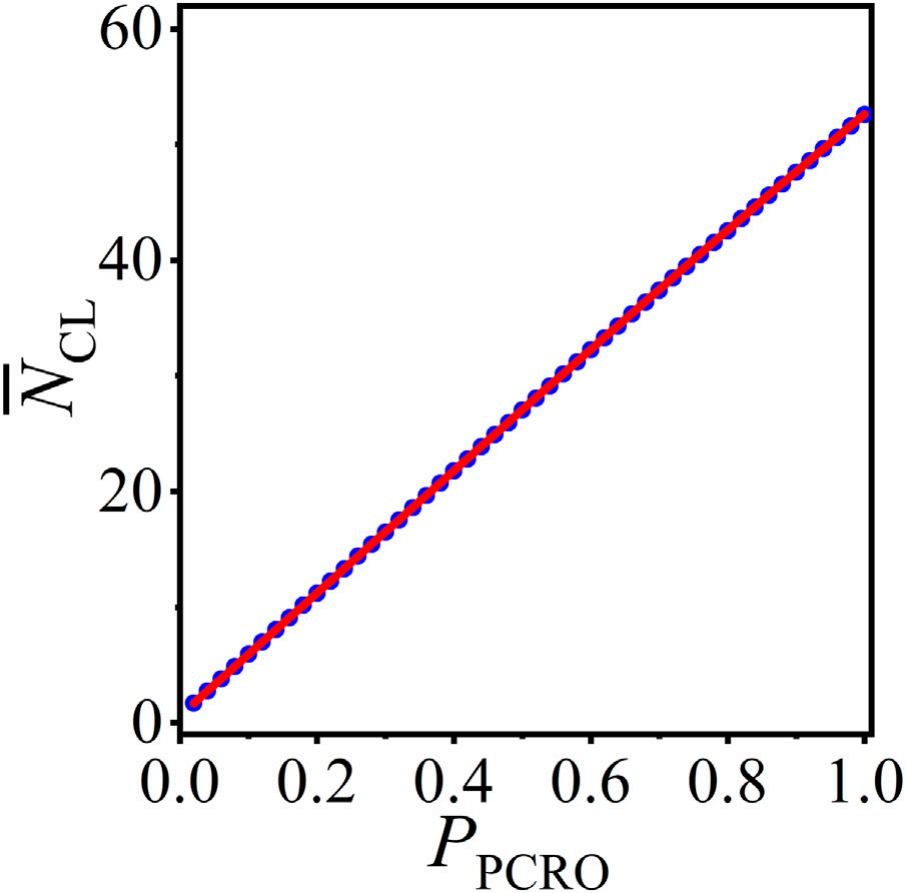
(Color online) The numerical results (blue circles) and theoretical predictions (red curves) of the average number of CLs ${\bar{N}}_{CL}$ in the operated ERRNs with PCRO probability $P_{PCRO}$ .

Based on the above results, we can now apply the statistical analysis method to forecast the average number of CLs in the operated ERRN, which are formed in the cutting and rewiring procedures of the PCRO and largely determined by the PCRO probability condition. Figure 5 presents the numerical results (blue circles) and theoretical predictions (red curves) of the average number of CLs ${\bar{N}}_{CL}$ in the operated ERRN on the PCRO probability $P_{PCRO}$ . The numerical and theoretical numbers of CLs both increase significantly as the PCRO probability increases, confirming not only the effectiveness of the statistical analysis method proposed here but also the effects of the PCRO in regulating the structure of the given network. At this point, the validity and applicability of the statistical analysis method have been verified in the ERRNs with a PCRO.

## 6 The universality of the statistical analysis method

**FIGURE 6:**
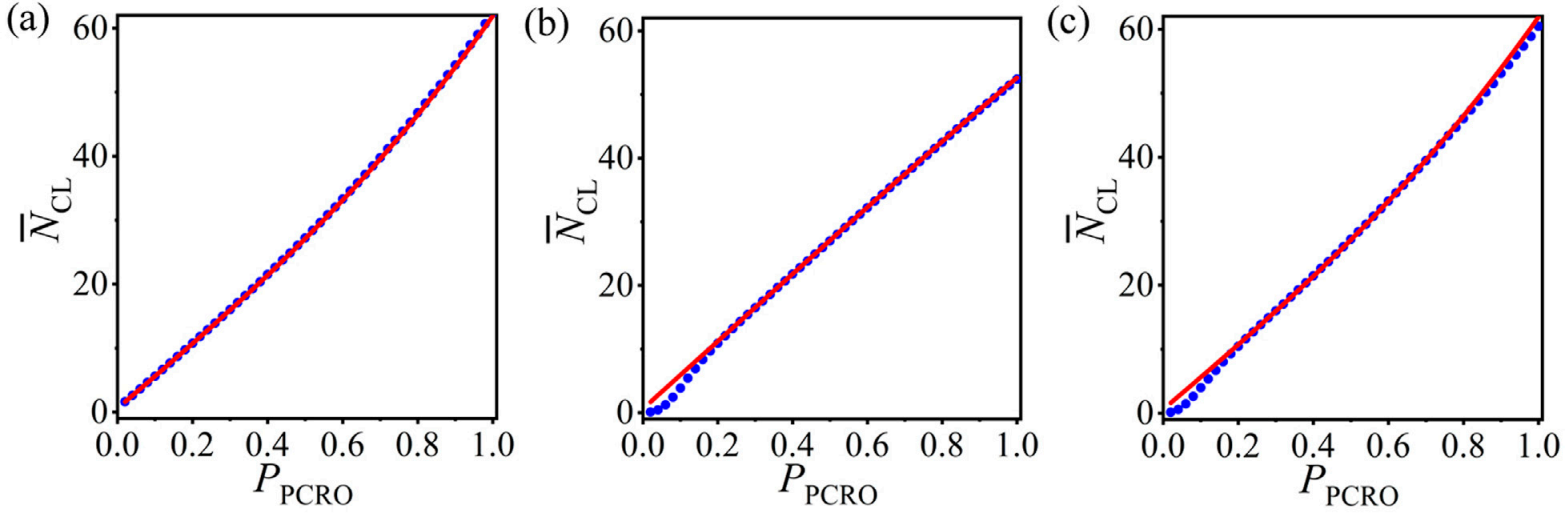
(Color online) The numerical results (blue circles) and theoretical predictions (red curves) of the average number of CLs ${\bar{N}}_{CL}$ in the operated ERRNs for other preferential constraints in the PCRO cutting and rewiring procedures. **(A)** $k_{1}={\left\langle k\right\rangle }_{AV}$ , $k_{2}=k_{Max}$ , and $k_{3}=k_{Max}^{\prime }$ ; **(B)** $k_{1}=1$ , $k_{2}={\left\langle k\right\rangle }_{AV}$ , and $k_{3}={\left\langle k\right\rangle }_{AV}^{\prime }$ ; **(C)** $k_{1}={\left\langle k\right\rangle }_{AV}$ , $k_{2}=k_{Max}$ , and $k_{3}={\left\langle k\right\rangle }_{AV}^{\prime }$ . Here, ${\left\langle k\right\rangle }_{AV}$ and ${\left\langle k\right\rangle }_{AV}^{\prime }$ present the actual values of average degree in the networks before and after the cutting procedure, respectively. $k_{Max}$ and $k_{Max}^{\prime }$ are the maximum degrees in the corresponding networks, respectively.

It is necessary to inspect the universality of our statistical analysis method. The average number of CLs ${\bar{N}}_{CL}$ in the operated ERRNs with a PCRO is still utilized as an example to explore this issue. Based on the preferential constraints and the network structure parameters utilized in Figure 2, we first test our method with other preferential constraints in the PCRO. The corresponding results are respectively displayed in Figure 6A ( $k_{1}={\left\langle k\right\rangle }_{AV}$ , $k_{2}=k_{Max}$ , and $k_{3}=k_{Max}^{\prime }$ , that is, only the preferential constraint in the cutting procedure is changed); Figure 6B ( $k_{1}=1$ , $k_{2}={\left\langle k\right\rangle }_{AV}$ , and $k_{3}={\left\langle k\right\rangle }_{AV}^{\prime }$ , that is, only the preferential constraint in the rewiring procedure is changed); and Figure 6C ( $k_{1}={\left\langle k\right\rangle }_{AV}$ , $k_{2}=k_{Max}$ , $k_{3}={\left\langle k\right\rangle }_{AV}^{\prime }$ , that is, both the preferential constraints in the cutting and rewiring procedures are changed). Here, ${\left\langle k\right\rangle }_{AV}$ and ${\left\langle k\right\rangle }_{AV}^{\prime }$ present the actual values of the average degree in the network before and after the cutting procedure. $k_{Max}$ and $k_{Max}^{\prime }$ are the maximum degrees in the corresponding network. The theoretical predictions (red curves) revealed with other preferential constraints coincide well with the numerical results (blue circles). This confirms that the statistical analysis method proposed here is irrelevant to the preferential constraints in the PCRO.

**FIGURE 7:**
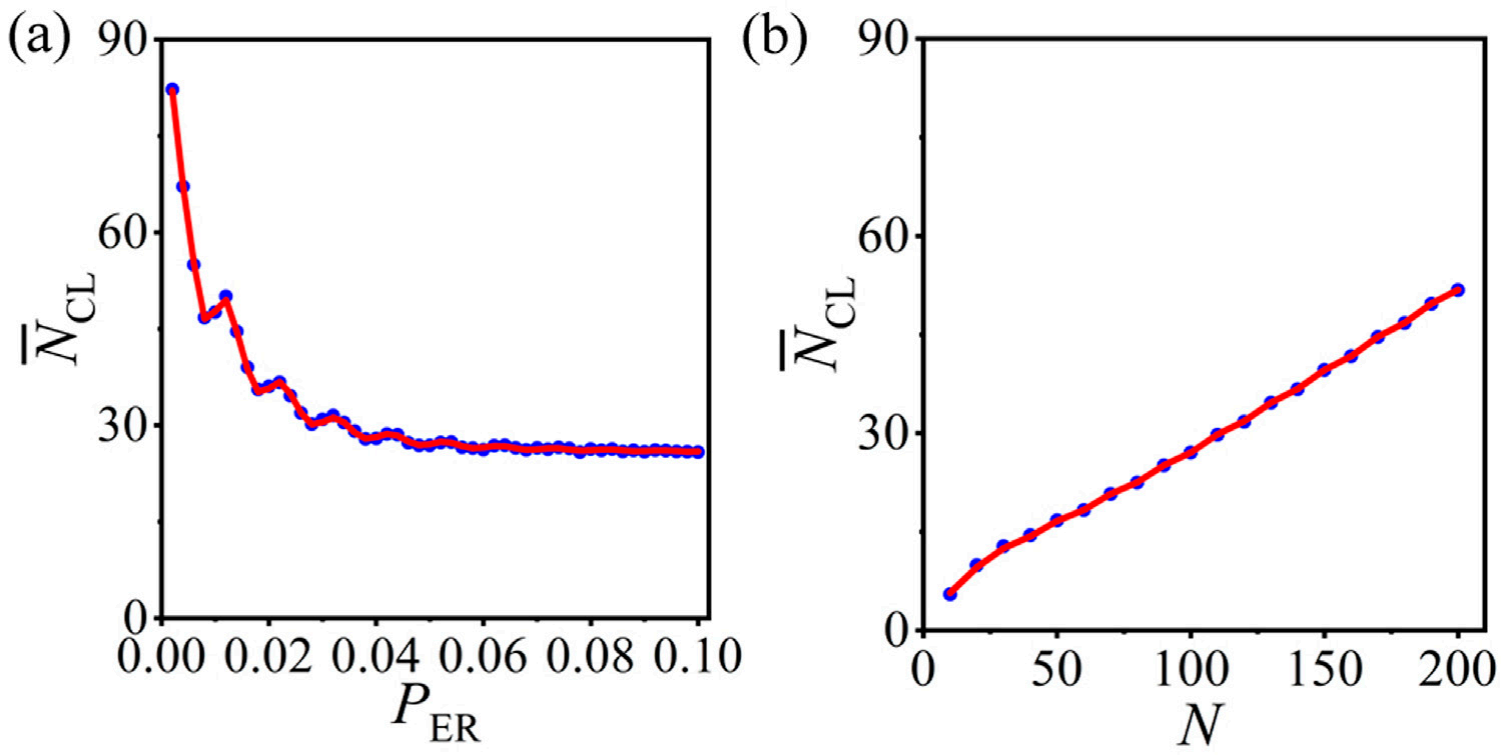
(Color online) The numerical results (blue circles) and theoretical predictions (red curves) of the average number of CLs ${\bar{N}}_{CL}$ in the operated ERRNs for other connection probabilities $P_{ER}$ [**(A)**] and system sizes N [**(B)**]. The same preferential constraints used in Figure 2 and the PCRO probability $P_{PCRO}=0.50$ are utilized here.

The statistical analysis method is applicable to general ERRN structures. The preferential constraints are the same as those in Figure 2, and the PCRO probability $P_{PCRO}=0.50$ is employed for the following discussion. The theoretical predictions (red curves) and the numerical results (blue circles) obtained for other different connection probabilities $P_{ER}$ and system sizes N are displayed in Figures 7A, B, respectively. Note good alignment between theoretical predictions and numerical results. This further verifies the universality of our statistical analysis method. Here, we should also mention that the idea of the theoretical deduction can be extended and applied to other paradigmatic network models, such as homogeneous random networks, small-world networks, and even scale-free networks. However, the corresponding formulas and conclusions may not be the same.

## 7 Mimicking epileptic-seizure-related synchronization phenomena in brain systems

Modeling specific physiological processes and physiological functions from the perspective of network physiology should be discussed. In this part, we use the PCRO method proposed in this article to mimic the epileptic-seizure-related synchronization phenomena in pathological brain systems. This issue was first studied from the perspective of network physiology by Gerster et al. (2020) and Schöll (2021). They revealed that, in addition to the empirical brain network, the small-world networks with intermediate rewiring probability can also reproduce the epileptic-seizure-related synchronization phenomena that closely resemble the ones seen during epileptic seizures in humans (see Figure 7 in Gerster et al. (2020) and Figure 3 in Schöll (2021)). In this case, the corresponding network structure properties are found to be C=0.25 (the average clustering coefficient) and L=3.0 (the mean shortest path length), by which the balance of regularity and randomness of the given network is revealed. The authors claimed that the network topology with a certain balance of regularity and randomness is the key factor in determining the self-initiation and self-termination of episodes of seizure-like synchronization.

Based on the discussions presented in the above sections, we can conclude that the proposed PCRO method also has the effect of regulating the network structure. By applying the PCRO to the paradigmatic network models, the corresponding structures will undergo a transition from homogeneous to heterogeneous. So we would ask whether a similar balance of regularity and randomness can also be induced by the PCRO, by which the same epileptic-seizure-related synchronization phenomena can be mimicked by our scenario.

**FIGURE 8:**
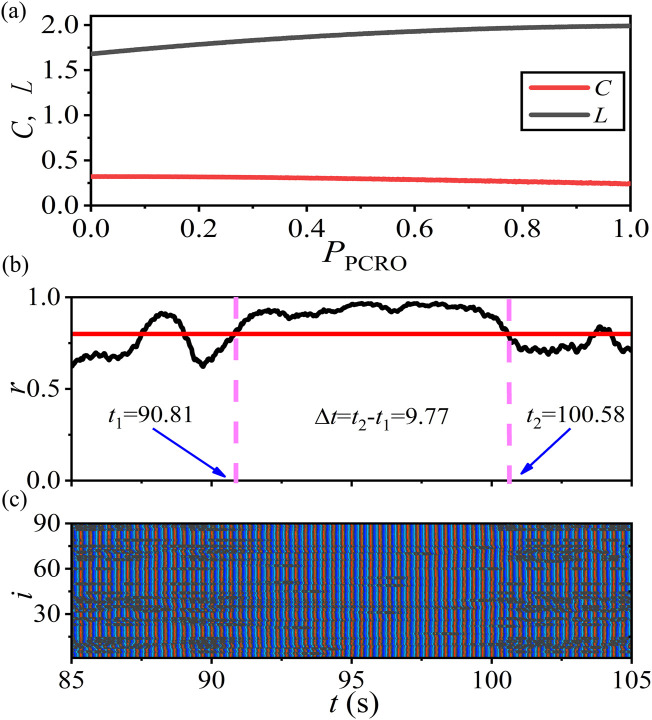
(Color online) Mimicking the epileptic-seizure-related synchronization phenomena in brain systems by the PCRO method proposed in this article. Here, an ERRN consisting of FitzHugh–Nagumo (FHN) neurons with the rotational coupling scheme is utilized to address this issue. The system size (N=90) and the parameter setting of the FHN network ( α=0.5 , ε=0.05 , and $\varphi =\frac{\pi }{2}-0.1$ ) are all the same as the ones adopted by Gerster et al. (2020) and Schöll (2021), except for the coupling strength σ=0.07 . The connection probability of the initial ERRN is chosen as $P_{ER}=0.032$ . **(A)** The dependence of the average clustering coefficient C (red line) and the mean shortest path length L (black line) of the operated FHN network on the PCRO probability $P_{PCRO}$ . The preferential constraints are selected as $k_{1}=1$ , $k_{2}={\left\langle k\right\rangle }_{AV}$ , and $k_{3}=k_{Max}^{\prime }$ . **(B)** The corresponding global Kuramoto order parameter r (black curve) vs. time relative to the onset of a seizure (time interval 20 s) obtained at PCRO probability $P_{PCRO}=0.97$ . The horizontal red line marks the threshold of r=0.8 . If r>0.8 for more than 8 s, an epileptic-seizure-related synchronization can be identified on the FHN network with a PCRO. The two vertical pink dashed lines, respectively, indicate the instants of the self-initiation and the self-termination of episodes of seizure-like synchronization. **(C)** The space-time plot of the dynamical phases corresponding to panel **(B)** by which the PCRO method induced the epileptic-seizure-related synchronization is further confirmed.

An ERRN consisting of FitzHugh–Nagumo (FHN) neurons with the rotational coupling scheme is utilized to address this issue. The system size (N=90) and the parameter setting of the FHN network ( α=0.5 , ε=0.05 , and $\varphi =\frac{\pi }{2}-0.1$ ) are all same as the ones adopted by Gerster et al. (2020) and Schöll (2021) except for the coupling strength σ=0.07 . The connection probability of the initial ERRN is chosen as $P_{ER}=0.032$ . Figure 8A first displays the dependence of the average clustering coefficient C (red line) and the mean shortest path length L (black line) of the operated FHN network on the PCRO probability $P_{PCRO}$ . The preferential constraints are selected as $k_{1}=1$ , $k_{2}={\left\langle k\right\rangle }_{AV}$ , and $k_{3}=k_{Max}^{\prime }$ . It is shown that, as the PCRO probability increases, the average clustering coefficient decreases gradually, while the mean shortest path length increases. Importantly, as $P_{PCRO}$ approaches 1.0, the approximate network structure properties of C and L are those for the emergence of epileptic-seizure-related synchronization on the small-world network with intermediate rewiring probability can be obtained. This means that, in these parameter regions, our PCRO method can also induce a certain balance of regularity and randomness on the operated FHN network, based on which the corresponding epileptic-seizure-related synchronization phenomena are expected to be observed.

The global Kuramoto order parameter r (black curve) of the operated FHN network obtained at a PCRO probability of $P_{PCRO}=0.97$ is revealed in Figure 8B. The horizontal red line marks the threshold of r=0.8 . The definition of the epileptic-seizure-related synchronization, which was first introduced by Schöll et al., is also utilized here; that is, the global Kuramoto order parameter should satisfy r>0.8 for more than 8 s. The two vertical pink dashed lines, respectively, indicate the instants of the self-initiation and the self-termination of episodes of seizure-like synchronization, between which an epileptic-seizure-related synchronization can be identified on the FHN network with a PCRO. Figure 8C shows the corresponding space-time plot of the dynamical phases by which the PCRO method that induced the epileptic-seizure-related synchronization is further verified. These results can confirm the possible application of the PCRO method proposed in this article in mimicking specific physiological phenomena in real cases.

## 8 Conclusion

In conclusion, a *preferentially cutting–rewiring operation* is proposed in the present article to regulate the structure of the given network. It consists of two distinct stages: the cutting procedure and the rewiring procedure with specific preferential constraints. By applying the PCRO on the classical ERRN with specific constraints and a certain PCRO probability, the initially homogeneous structure changes drastically. Three types of isolated nodes are generated: the NINs, the ADINs, and the PDINs, based on which the CLs are formed between the two hubs in the operated network. Furthermore, as the PCRO probability increases, the number of CLs increases significantly, which makes the initially homogeneous ERRN become heterogeneous. This confirms that the PCRO introduced in this article has effects on regulating the network structure.

The statistical properties of the ERRN with a PCRO are theoretically studied using a statistical analysis method. We have analytically derived the statistical expressions of the probability distributions of NINs, ADINs, and PDINs, based on which the probability distribution of CLs is acquired easily. More importantly, the theoretical predictions obtained from these analytical formulas have been confirmed in numerical simulations and coincide with the experimental data very well. Furthermore, these analytical expressions are applied to forecast the average number of CLs in the operated ERRN. The coincidence of the numerical and theoretical results confirms the validity and applicability of the statistical analysis method proposed here. Finally, the universality of the statistical analysis method has also been verified. Our method is general and can be applied to ERRNs with arbitrary preferential constraints and topologies.

Modeling specific physiological processes and physiological functions from the perspective of network physiology is an important and central issue under investigation in the interdisciplinary field of complexity science and biological science. The PCRO method proposed in this article, which consists of the cutting procedure and the rewiring procedure, may give us a clue in understanding the physiological process of the global information integration among the localized functional modules in structural and functional brain networks to implement specific physiological functions. The reasons are as follows. In the PCRO cutting procedure, we discard the links of the nodes satisfying the preferential constraint with a certain PCRO probability condition, and isolated nodes are produced in the original network. As these isolated nodes originally satisfied the given preferential attribute, they can be roughly considered the localized functional modules in anatomical space, which are selected and will be integrated into achieving global information communication across the whole brain system for specific physiological functions. In the PCRO rewiring procedure, we reconnect these isolated nodes to the hubs satisfying the corresponding preferential constraint, which can be roughly regarded as the specialized regulatory centers (i.e., the hub regions) in brain networks to perform global physiological functions among the whole brain system. Furthermore, in a recent contribution (Qian et al., 2024b), the PCRO-induced oscillation mode transition from the originally single-mode oscillations to the newly multi-mode oscillations has been confirmed to emerge among the preferentially operated nodes (i.e., the integrated local modules), which we think is beneficial for understanding the complicated global multimodal physiological functions in integrated structural and functional brain networks. More importantly, the probability distributions of the three different types of isolated nodes formed in the PCRO are derived according to the statistical analysis method proposed in this article, based on which the probability distribution of the preferentially operated common leaves is acquired explicitly. We think the statistical analysis method and the precise theoretical formulas exposed in this article can shed light on a deep comprehension of these amazing physiological phenomena in highly complex and heterogeneous brain networks. We do hope our results will be of great interest to network physiology.

## Data Availability

The raw data supporting the conclusions of this article will be made available by the authors, without undue reservation.
